# The effect of educational application in nursing internship clinical training on cognitive and functional skills and students’ satisfaction

**DOI:** 10.1186/s12912-024-01954-5

**Published:** 2024-06-05

**Authors:** Maryam Khoshbakht-Pishkhani, Nazila Javadi-Pashaki, Niloufar Asgharzadeh Esfandi, Masoomeh Bagheri Koodakani, Saman Maroufizadeh, Ali Hamidi Madani

**Affiliations:** 1 Department of Medical Surgical Nursing, School of Nursing and Midwifery, Guilan University of Medical Sciences, Rasht, Iran; 2 Medical Education Research Center (MERC), Guilan University of Medical Sciences, Rasht, Iran; 3 Social Determinants of Health Research Center (SDHRC), Guilan University of Medical Sciences, Rasht, Iran; 4 Student Research Committee, School of Nursing and Midwifery, Guilan University of Medical Sciences, Rasht, Iran; 5 Department of Biostatistics and Epidemiology, School of Health, Guilan University of Medical Sciences, Rasht, Iran; 6 Urology Research Center, School of Medicine, Razi Hospital, Guilan University of Medical Sciences, Rasht, Iran; 7 Guilan University of Medical Sciences, Guilan University of Medical Sciences, Rasht, Iran

**Keywords:** Application, Clinical education, Nursing, Traditional education, Electronic education

## Abstract

**Background:**

Clinical education plays an essential role in shaping the nursing identity and is one of the central elements in the education of nursing students. Today, with the advancement of novel technologies, utilizing mobile phone-based technologies in the education of medical sciences is inevitable. Therefore, this study was conducted with the aim of investigating the impact of the urology educational application on nursing students’ cognitive-functional criteria and satisfaction during the internship period.

**Methods:**

This experimental educational intervention study was conducted during nursing students’ urology internship course at Shahid Beheshti School of Nursing and Midwifery in Rasht. The data collection tools included a demographic characteristics questionnaire, cognitive skills scale, functional skills scale, and satisfaction scale (Stokes, 2001). The data were analyzed using SPSS software version 16, and a significance level was set at 0.05.

**Results:**

Out of 48 studied students, 28 (58.3%) were males. The mean age of the students was 20.34 (SD = 1.51) years. In the application group, the mean of students’ cognitive skills after the intervention significantly increased by 2.33 units (95% CI: 1.73 to 2.9) (t_(23)_ = 7.97, *P* < 0.001, d = 1.626). By controlling the scores before the intervention, the adjusted mean score of cognitive skills in the application group was 0.56 units (95% CI: -0.16 to 1.28) higher than the traditional group; however, this difference was not statistically significant (F_(1, 45)_ = 2.42, *P* = 0.127, η^2^_p_ = 0.051). There was no statistically significant difference between the mean score of students’ functional skills in traditional and application groups (t_(46)_ = 0.63, *P* = 0.532, d = 0.184). The total mean score of satisfaction with education in the application group was 83.0 (SD: 10.7). According to the values ​​of the quartiles, 75% of the students scored higher than 75.9, 50% scored higher than 83.9, and 25% scored higher than 91.1.

**Conclusion:**

According to the results of this study, students’ scores of functional and cognitive assessment and satisfaction with the application in urology clinical training were reported as favorable. Therefore, it is recommended that mobile phone-based technologies be used in students’ clinical education and internships in combination with the traditional method.

**Supplementary Information:**

The online version contains supplementary material available at 10.1186/s12912-024-01954-5.

## Background

Nursing clinical education provides opportunities for students to practice and repeat clinical scenarios [1]. Every clinical experience is not merely for acquiring a specific practical knowledge or skill. Rather, clinical training and experiences should improve nursing students’ self-efficacy and change them to those with the ability to provide high-quality care in clinical environments [2]. Different traditional and modern methods are used in nursing education. The traditional teaching method that has been used for a long has known advantages and disadvantages. One advantage of this method is that it helps encourage learning and establish a connection between past and present experiences in learners, and as a result, it facilitates and accelerates the acquisition of information and receiving feedback [3]. Its disadvantages include reducing the opportunity for thinking in students, forgetting more of the trained material short after teaching, and encouraging the learner to learn passively. Moreover, inattention to learners’ individual differences and needs, problem-solving, creative thinking, and other high-level cognitive skills and their low effectiveness can be mentioned as disadvantages [4].

Considering the changes and technological advances in the last few decades and the need to use innovative educational methods in various fields, including medical sciences, it seems inevitable to revise the conventional approaches [5]. Therefore, since the main goal of nursing education is to train skilled and qualified nurses [6], nursing and its education should also be adapted to these changes. Theorists believe that training through traditional methods is no longer practical due to their dependence on specific times and places. Consequently, in order to maintain its advancement, nursing educators must use modern educational methods, which significantly increase students’ learning by preventing their superficial learning [5]. In this regard, adopting approaches that lead to more student participation in the learning process can facilitate the presentation of course content. One of these solutions is the use of novel and electronic technologies in education. Since the potential of electronic education using new and flexible approaches in nursing education is known at the international level [7, 8], electronic learning, which is rapidly growing, can be considered an alternative method to providing education [9].

In line with the inclination towards electronic education, smartphones have become indispensable for nursing students. Besides, using these devices in the clinical environment can have distinct impacts on the nursing students’ clinical practice [10, 11]. The results of the studies indicate that smartphone-based learning has significant positive influences on nursing students’ knowledge, skills, self-assurance, and learning attitude, suggesting that smartphone-based mobile learning might become an alternative or supporting method for better education in the nursing field [12–14]. However, despite the advantages of training through smartphones, several studies indicate that using mobile phones in the clinical setting can jeopardize patient safety due to distraction and disruption in clinical care [10]. On the other hand, in a study conducted to perceive undergraduate nursing students’ opinions regarding the use of smartphones and mobile applications, nursing students reported numerous benefits of mobile phone technology, such as better access to educational materials, improved knowledge and self-confidence, and reduced level of anxiety about clinical learning [15, 16].

Therefore, given the contradictory results in the studies and the significant role of clinical education in the formation of nursing students’ clinical knowledge, as well as the students’ enthusiasm to use smartphones and mobile applications as educational tools in clinical environments, this study aimed to compare the traditional method and the mobile application on students’ cognitive-functional criteria and satisfaction during the urology credit of the internship course in Rasht School of Nursing and Midwifery, and the study hypothesis is that students who receive the urology educational application during the internship period may have higher mean cognitive and functional skills at posttest measurement compared to the control group.

## Methods

This experimental educational intervention study was carried out with the aim of determining the effect of the educational application on undergraduate nursing students’ cognitive-functional criteria and satisfaction with the urology internship course. Since in the nursing curriculum, urology internship credit is planned for nursing students in the 4th semester, and the researcher noticed the learning problems of the students in this unit; therefore, this semester was selected to conduct the research. The inclusion criteria for the 4th-semester nursing students included the willingness to participate in the study and possession of a smartphone. The exclusion criteria included failure to fully participating in the internship. A total of 48 students were randomly divided into intervention (24 students) and control groups (24 students). The internship course for each group was eight days (6 hours 8–13). Four groups, including six students entered the intervention, and four groups, including six students entered the control group). In the intervention group, the traditional method along with the application were used. On the first day of the internship, the application was provided to the students; they installed it, and the contents of the different parts of the application were explained to them. According to the lesson plan, at the beginning of the internship (8–9 am) and the end of each internship day (12:00–13:00.), in each day of the course, the educational topics of that day were reviewed by the students, and the students were asked to study the next day’s contents in the application. They could also use the application; however, they were asked not to use the phone during the administration of medication and the procedure at the bedside. In the control group, only the traditional method was used. The data collection tool included a questionnaire including demographic characteristics, an electronic questionnaire with ten questions about the cases and medications in the urology ward that assessed cognitive skills through pretest and post-test, in which each question was scored between 0–10, and a functional skills scale using a form to evaluate the internship course designed by the internal surgery group based on the nursing curriculum whose face and qualitative content validity had been established and was used as an approved evaluation form to evaluate students’ urology clinical internship. This form consisted of sections for assessment, accurate use of the urology ward terms, medications administration, performing various nursing care before and after the operation, performing diagnostic procedures and tests, providing care based on the nursing process, interpretation of specific tests, performing kidney and urinary tracts examinations, controlling vital signs and teaching to the patient during the internship by the clinical instructor. The Functional skill in each evaluated section was scored on a 5-point Likert scale from ‘Very good’ to ‘Very poor,’ and it was calculated as 0–50. A higher score indicated a higher Functional skill. Another tool was the Satisfaction Scale (adapted from Stokes, 2001), which was used in Zhonggen et al.’s study in 2019, and its validity and reliability were evaluated and confirmed [17]. The Satisfaction Scale consisted of 14 five-point Likert items, which evaluates the student’s satisfaction with the mobile phone-based learning method. Questions include: “My technology knowledge level is sufficient to learn through a mobile-based learning platform.’ ‘I am somewhat related to the educational environment by participating in a course that emphasizes learning through a mobile learning platform,’ ‘I prefer to take most of my classes through a mobile-based learning platform.’ In this tool, the higher the score, the higher the level of satisfaction. The face validity of the satisfaction scale was investigated in 15 students. In addition, the questions’ clarity, simplicity, and comprehensibility were investigated and approved. The qualitative content validity of the investigated tools was also evaluated using the opinions of ten professors and clinical instructors, and their opinions were applied. The reliability of the scale for satisfaction with the application was investigated in 15 students, and Cronbach’s alpha was reported as 0.85. (All scales and evaluation forms attached)

### Descriptions of the application for the urology internship course

This application was designed and developed by a health information management engineer who was also a nursing expert using the Appeto app builder(https://appeto.ir). The educational content was extracted from nursing and medical reference books by an undergraduate nursing student under the supervision of a nursing faculty member who taught the subject under study theoretically and practically. Moreover, two nursing faculty members and a urology expert reviewed, modified and approved its content validity. In the application, each internship day’s lesson plan and program were organized based on the urology internship logbook, and the contents taught each day were placed in the relevant ward based on the schedule. The contents were based on the clinical lesson plan of the urology ward internship, which included ward-related terms, examinations of the kidney system and urinary tracts, familiarization with kidney and urinary tract diseases and related nursing care, medications of the urology ward, diagnostic and therapeutic procedures and operations of the urology ward, and valuable materials for working with the ward’s equipment. By using the application, each student would click on the specified program and enter that day’s content according to the specified course plan. At the beginning of the course, a pretest was placed through the Google form link and activated in the app in the evaluation section. Students completed the test in ten minutes at the beginning of the internship, and the answers were emailed to the clinical instructor. The videos and content related to each internship day were placed in a specific section. The students entered the relevant page of application and reviewed the content at the beginning and the end of each internship day (8–9 a.m. and 12 − 1 p.m.) when it did not interfere with other tasks related to the internship course. Every day before the internship, the topics and practices of the previous day were reviewed, and the tasks planned during the current day were implemented by the students using the application. At the end of the internship, students were also tested for functional, cognitive skills and satisfaction.

### Sample size

At first, the sample size calculation was performed for an independent t test using G*Power version 3.1.9.2 [18]. Since we planned to incorporate the pretest scores in the statistical analysis using analysis of covariance (ANCOVA), we multiplied this number by a design factor of (1- ρ2), where ρ is the correlation coefficient between the pretest and posttest scores [19]. With an effect size of 0.8 (Cohen’s d) for cognitive skills score, a power of 0.8, an alpha value of 0.05, and using *r* = 0.5 to account for the correlation between pretest and posttest scores, 20 nursing students would be required in each group. Assuming a potential drop-out rate of 15%, 24 students were required in each group (total sample size = 48).

### Statistical analysis

In this study, the values of quantitative and qualitative variables are presented as ‘mean (standard deviation)’ and ‘frequency(percentage),’ respectively. Paired t-test was used to compare the mean score of nursing students’ cognitive skills before and after the intervention for traditional and application groups. The mean score of cognitive skills was compared between the two traditional and application groups after the intervention using analysis of covariance (ANCOVA). The mean scores of functional skills were compared in the traditional and application groups after the intervention using an independent t-test. In addition, Cohen’s d effect size was reported for the paired t-test, an independent t-test, as well as partial ETA squared (η^2^_p_) for ANCOVA. η^2^_p_ values ​​equal to 0.01–0.06, 0.06–0.14, and > 0.14 indicate small, medium, and large effect sizes, respectively. For Cohen’s d, values ​​of 0.2–0.5, 0.5–0.8, and > 0.8 indicate small, medium, and large effect sizes, respectively [20]. The data were analyzed using SPSS software version 16, and a significance level was set at 0.05.

## Results

### Nursing students’ individual and educational characteristics

The individual and educational characteristics of the studied students are shown in Table 1. The mean age of the students was 20.34 (SD = 1.51) years. Out of 48 studied students, 28 (58.3%) were males.

**Table 1:** Nursing students’ individual-educational characteristics in traditional and application groups

variable	Total (*n* = 48)	Group	
		Traditional (*n* = 24)	Application (*n* = 24)
Age (years), mean (SD)	20.34 (1.51)	20.45 (1.68)	20.23 (1.35)
Sex, n (%)			
Male	28 (58.3)	15 (62.5)	13 (54.2)
Female	20 (41.7)	9 (337.5)	11 (45.8)

### Comparison of students’ cognitive skills scores before and after the intervention in the two groups

**Fig. 1:**
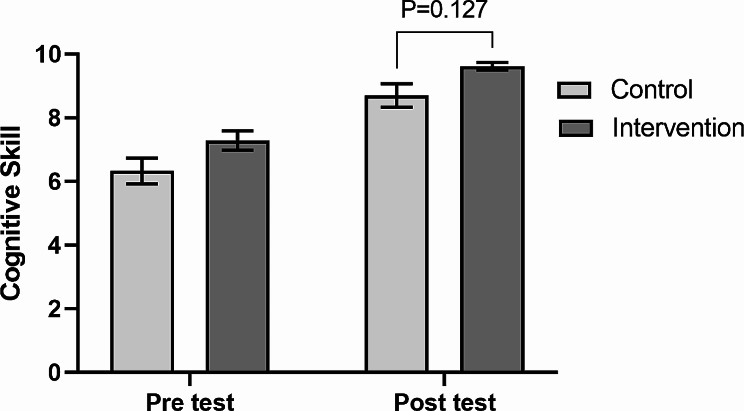
Comparison of students’ cognitive skills scores after the intervention between traditional and application groups. Values are presented as ‘mean with 95% confidence interval.’ *P* value is based on ANCOVA

**Fig. 2:**
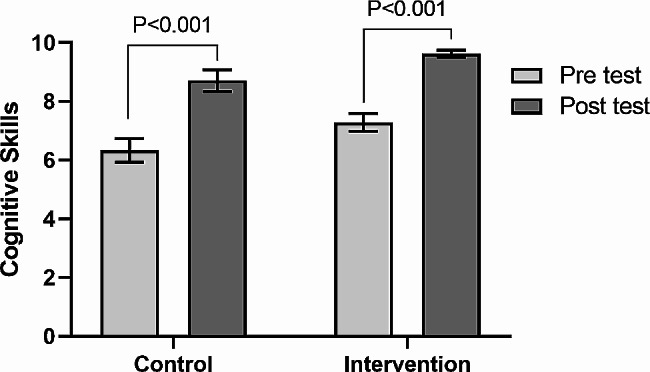
Comparison of students’ cognitive skills scores before and after the intervention by traditional and application groups. Values are presented as mean with a 95% confidence interval. P values are based on the paired t-test

In order to compare the mean score of students’ cognitive skills before and after the intervention for the two groups of traditional and application, a paired t-test was used. As can be seen in Table 2; Fig. 1, in the application group, the mean score of students’ cognitive skills after the intervention increased significantly by 2.33 units (95% CI: 1.73 to 2.94) (t_(23)_ = 7.97, *P* < 0.001, d = 1.626). Moreover, in the control group, the mean score of students’ cognitive skills after the intervention increased significantly by 2.38 units (95% CI: 1.62 to 3.13) (t_(23)_ = 6.5, *P* < 0.001, d = 1.328). The comparison of students’ cognitive skills scores before and after the intervention by traditional and application groups shows in Fig. 2.

**Table 2:** Comparison of students’ cognitive skills mean scores before and after the intervention by traditional and application groups

Group	Time		Mean differences (95% CI)	t_(23)_	*P* ^†^	Cohen’s d
	Pretest	Posttest				
Traditional	6.33 (1.99)	8.71 (1.83)	2.38 (1.62 to 3.13)	6.50	< 0.001	1.328
Application	7.29 (1.49)	9.62 (0.58)	2.33 (1.73 to 2.94)	7.97	< 0.001	1.626

CI: Confidence Interval

† Paired t-test

Cohen’s d values of 0.2–0.5, 0.5–0.8, and > 0.8 indicate small, medium, and large effect sizes, respectively.

### Comparison of students’ cognitive skills score after intervention between the two groups

In Table 3, the mean scores of students’ cognitive skills after the intervention between traditional and application groups are compared using covariance analysis. By controlling the scores before the intervention, the adjusted mean score of cognitive skills in the application group was 0.56 units (95% CI: -0.16 to 1.28) higher than the traditional group; however, this difference was not statistically significant (F _(1,45)_ = 2.42, *P* = 0.127, η_p_^2^ = 0.051). The effect size of partial eta squared was 0.051, which was small. The Comparison of students’ cognitive skills scores after the intervention between traditional and application groups shows in Fig. 1.

**Table 3:** Comparison of students’ cognitive skills scores after the intervention between traditional and application groups

Time	Group		Adjusted mean difference ^a^ (95% CI)	ANCOVA results		
	Traditional	Application		F_(1, 45)_	*P*	η^2^_*p*_
Pretest	6.33 (1.99)	7.29 (1.49)				
Posttest	8.71 (1.83)	9.62 (0.58)	0.56 (-0.16 to 1.28)	2.42	0.127	0.051

CI: confidence interval; ANCOVA: analysis of covariance

^a^ Adjusted for values before the intervention

Partial ETA squared values (η^2^_p_) of 0.01–0.06, 0.06–0.14, and > 0.14 indicate small, medium, and large effect sizes, respectively.

### Comparison of students’ functional skills scores after intervention between the two groups

**Fig. 3:**
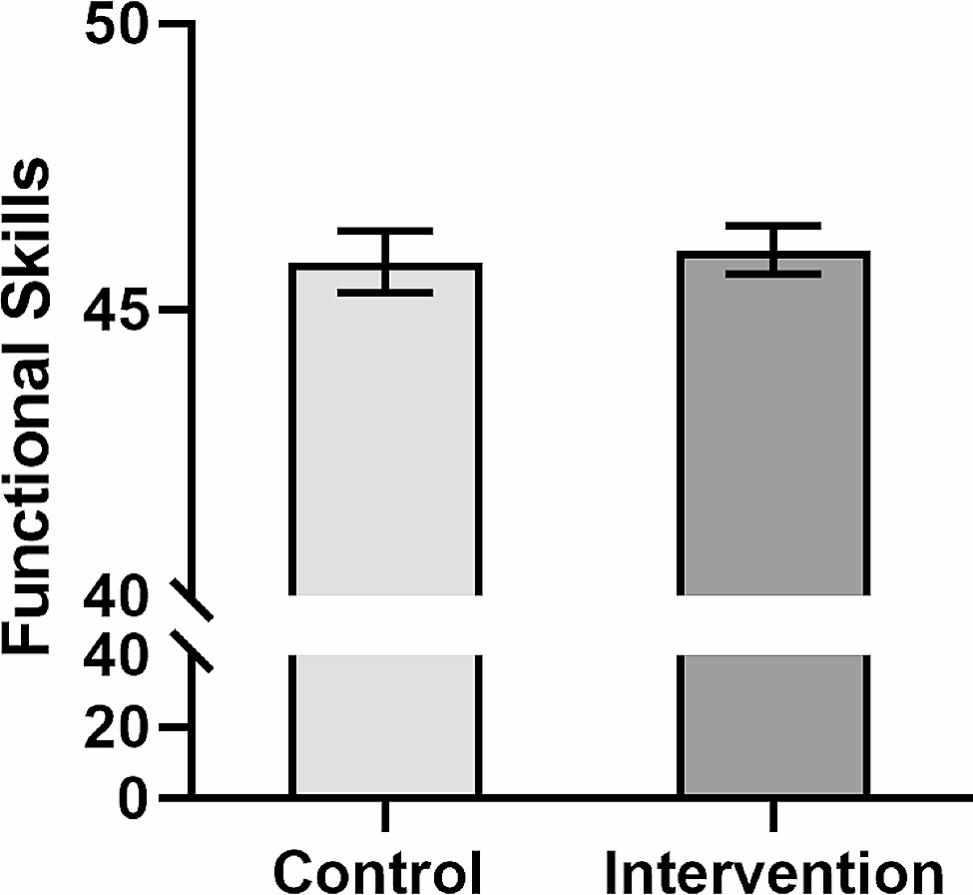
Comparison of students’ functional skill scores after intervention between traditional and application groups. Values are presented as ‘mean with 95% confidence interval.’ *P* value is based on independent t-test

Independent t-test was used to compare the mean score of students’ functional skills after the intervention between traditional and application groups. As can be seen in Table 4, there is no statistically significant difference between the mean score of students’ functional skills in traditional and application groups (t_(46)_ = 0.63, *P* = 0.532, d = 0.184). the Comparison of students’ functional skill scores after intervention between traditional and application groups shows in Fig. 3.

**Table 4:** Comparison of the mean score of students’ functional skills after intervention between traditional and application groups

	Group		mean difference (95% CI)	t _(46)_	*P* ^†^	Cohen’s d
	Traditional	Application				
Functional skill score	45.83 (1.27)	46.04 (1.00)	0.21 (-0.46 to 0.87)	0.63	0.532	0.184

CI: Confidence Interval

^†^ Independent t-test

Cohen’s d values of 0.2–0.5, 0.5–0.8, and > 0.8 indicate small, medium, and large effect sizes, respectively.

### Satisfaction with training in the application group

**Fig. 4:**
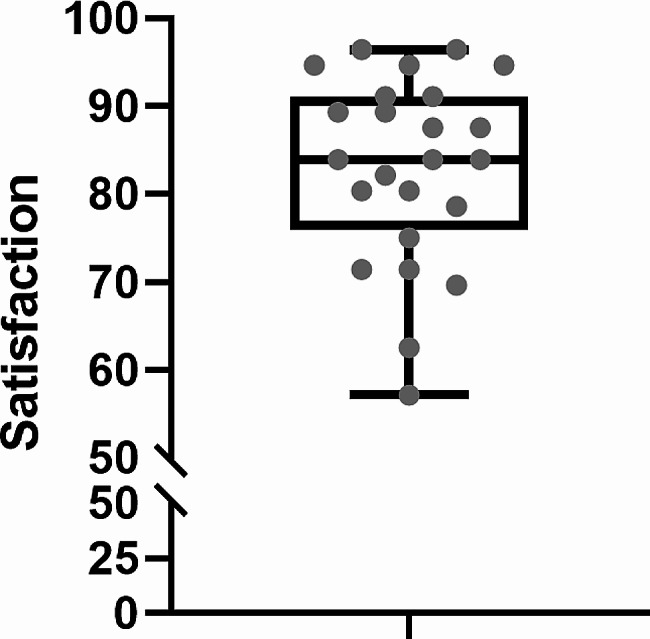
Box plot of the total score of satisfaction with training in the application group. Note. In the box plot, minimum, first quartile, median, third quartile, and maximum values are presented, respectively

The mean total score of satisfaction with training in the application group was 83.0 (SD: 10.7), and the median score was 83.9 (interquartile range: 75.9 to 91.1). According to the values ​​of the quartiles, 75% of students scored higher than 75.9, 50% scored higher than 83.9, and 25% scored higher than 91.1 (Fig. 4).

The possible range of total satisfaction scores is 0-100.

## Discussion

Nursing students face various challenges to learn in the clinical environment. Considering this, mobile phone-based technology can be beneficial in dealing with these challenges (,17,21). In this study, an application and its effect on students’ cognitive criteria and satisfaction with training in urology nursing internship were compared to the traditional method. The students were assigned into two groups. In one group, the traditional internship method with the presence of a trainer and practicing with cases, files, and patients, as well as providing care based on the conventional lesson plan, was implemented. However, in the second group, the application was used along with the traditional method of internship training. The results of the present study showed that the cognitive skills in the application group were higher than those of the traditional group; however, the difference was not significant. It seems that using the application improved the students’ cognitive skills more than the traditional method. In the study by Kim et al., conducted using a smartphone application to train nursing students in caring for airway obstruction in newborns, students’ knowledge and cognition scores in the experimental group were higher than those of the control group. Despite the lack of statistical difference, this study showed that smartphone-based training could be effective in nursing education related to airway obstruction in infants [22]. In this study, airway obstruction in infants was taught via video display on smartphones for the intervention group and as lecture-based traditional training for the control group, which was roughly similar to our study method. In the urology application, educational video links were used to improve students’ learning. Alikarami et al.’s study likewise reported the positive effect of the application on nursing students’ awareness of arterial blood gas interpretation in the hospital [13]. Therefore, mobile phone applications can be used as a complementary method along with conventional methods in clinical education to improve nursing students’ awareness.

Regarding functional skill scores, in this study, students’ scores in both groups were high after the educational intervention; however, they were slightly higher in the application group than in the traditional group. Nevertheless, there was no statistically significant difference between the mean score of students’ functional skills in the traditional and application groups after the intervention. This result indicates that due to the clinical nature of the course and students’ dealings with clinical cases in both methods and their practice and repetition with the help of the instructor, they were able to acquire functional skills at a high level, and the instructor taught functional skills perfectly in both methods. In Kim et al.’s study, nursing functional skills increased significantly in the experimental group after the intervention. In the present study, the pretest for functional criteria was not implemented in the two groups due to the effectiveness of training on these criteria, and they were evaluated after training methods were applied [22]. In the study by O’Connor & Andrews, students stated that they used smartphones less frequently to help them learn clinical practice; however, mobile technology helped them better access educational materials, improve knowledge, gain self-confidence, and reduce anxiety in clinical learning [21]. Using mobile phone applications in nursing education is a learner-oriented interactive method and an effective way to experience practical nursing skills. Therefore, it is recommended to design and use a mobile phone application with nursing education content that can be used effectively in all groups of nursing students [23].

According to the results of this study, the mean score of total satisfaction with education in the application group was high, and the students considered the use of the application as very desirable. Previous studies show that mobile phone applications in nursing education increase students’ self-confidence and competence and reduce stress. In addition, students believe that using both methods (the instructor’s lecture and mobile phone applications) simultaneously is highly practical. In line with these results, it is believed that the development of mobile phone applications for nursing education, their integration into traditional education, and the revision of curricula based on modern educational methods will be beneficial. Based on the results of the study conducted by Sheikhaboumasoudi et al. in 2018 in this field, nursing students obtained satisfactory results with a combination of electronic learning and traditional methods. Moreover, this study suggests that a combination of traditional methods with electronic education methods, such as using educational websites and interactive online resources for teaching basic nursing courses, can be an effective supplement for improving nursing students’ clinical skills [8].

In O’Connor & Andrews’s study, nursing students reported numerous benefits of mobile phone technology, such as better access to educational materials, improved knowledge and confidence, and reduced anxiety about learning in practice. Barriers such as nursing personnel’s negative attitudes, poor Wi-Fi connectivity, and the quality of educational content available in mobile phone applications were identified as some of the issues hindering mobile phone learning adoption in clinical nursing education. The results show that nursing students have a desirable perspective on mobile phone applications in clinical learning, and nursing schools should conduct more detailed research to improve the role of mobile phone technology in promoting clinical learning. The optimal way to personalize mobile phone applications is to meet students’ needs and ensure their usability at the bedside [21]. In the present study, the mobile phone application was used in the clinical training of the urology ward. Moreover, an attempt was made to provide the students with the necessary facilities to connect to the web-based content by providing the content in a suitable and usable platform through the application, as well as the internet password and a pocket Wi-Fi modem.

## Conclusion

Considering the favorable effect of using mobile phone technologies in students’ clinical education and their satisfaction with using new technology in education, mobile phone educational applications can be used to improve students’ cognitive and functional skills. The results of this study indicated that students’ scores on the functional and cognitive evaluation and their satisfaction with using the application in clinical urology training were favorable. Consequently, it is recommended that mobile phone-based technologies be used in combination with the traditional method in clinical training and internship courses. It is also suggested that more studies be conducted on the use of mobile phones in clinical training courses.

### Electronic supplementary material

Below is the link to the electronic supplementary material.

Supplementary Material 1

Supplementary Material 2

## Data Availability

All data and materials are available if needed.
