# Supplementary material for: The effect of educational application in nursing internship clinical training on cognitive and functional skills and students’ satisfaction
Source: BMC Nurs. 2024 Jun 5;23:381. doi: 10.1186/s12912-024-01954-5 (PMC11151607; doi:10.1186/s12912-024-01954-5)
Supplement: Supplementary file 1 — Supplementary Material 1 [file 12912_2024_1954_MOESM1_ESM.pdf]

Urology clinical unit evaluation form

(in Persian)

Name of the student:…………………………

Score:	Items:	Domain	Score:	Items:	Domain	
**Vary good (3), good (2.25),Moderate(1.5),Poor (0.75),very poor (0)**	10- Performs care before, during and after diagnostic procedures (cystoscopy, ureteroscopy, IVP, etc.)	**Nursing care in Diagnostic procedure's**	**Vary good (3), good (2.25),Moderate(1.5),Poor (0.75),very poor (0)**	1-Uses equipment correctly in ward	**Correct use of department equipment**	**(50) Evaluation of functional skills**
**Vary good (3), good (2.25),Moderate(1.5),Poor (0.75),very poor (0)**	11. Properly plans and implements the necessary care of clients suffering from urinary system disorders (infections, injuries, stones, tumors and abnormalities...) based on the nursing process .(4)	**Nursing care in patients**	**Vary good (3), good (2.25),Moderate(1.5),Poor (0.75),very poor (0)**	2-Uses medical terms and abbreviations related to urinary tract diseases correctly (2)	**Using the terminology of the urology department correctly**	
			**Vary good (3), good (2.25),Moderate(1.5),Poor (0.75),very poor (0)**	3-Infection control methods, including hand washing, proper disposal of contaminated and sharp objects, are fully used when caring for the patient	**Control of Infection**	
			**Vary good (3), good (2.25),Moderate(1.5),Poor (0.75),very poor (0)**	4- Observes the precautions related to safety and falls (bedside railing, wheelchair, transfer, movement) during the transfer and movement of the patient before and after surgery in the ward	**Patients safety**	
**Vary good (3), good (2.25),Moderate(1.5),Poor (0.75),very poor (0)**	12-Performs wound care, dressings, Foley catheters, and patient (drains correctly	**Wound care and dressing/drain and catheters**	**Vary good (3), good (2.25),Moderate(1.5),Poor (0.75),very poor (0)**	5- Performs the physical examination and taking the patient's history completely and correctly	**Physical Exam**	
**Vary good (3), good (2.25),Moderate(1.5),Poor (0.75),very poor (0)**	13. Plans and implements pre- and post-surgery nursing care of the patients under his care based on the nursing process	**Pre- and post-operative care**	**Vary good (3), good (2.25),Moderate(1.5),Poor (0.75),very poor (0)**	6- Performs I&O control in the patient correctly	**Control of I& O**	
**Vary good (3), good (2.25),Moderate(1.5),Poor (0.75),very poor (0)**	14. He/She provides necessary educations to his/her client (regarding drugs, diet, tests, etc.)	**Patient education**	**Vary good (4), good (3),Moderate(2),Poor (1),very poor (0)**	7- Controls and reports and charts the client's vital signs correctly	**Control of Vital Sign**	
**Vary good (3), good (2.25),Moderate(1.5),Poor (0.75),very poor (0)**	15 -He/she compiles a nursing report of his patient according to the correct principles of report writing.	**Nursing report**	**Vary good (3), good (2.25),Moderate(1.5),Poor (0.75),very poor (0)**	8-Collects the required laboratory samples (urine, fecal, blood, etc.) correctly	**Laboratory sampling**	
**Vary good (3), good (2.25),Moderate(1.5),Poor (0.75),very poor (0)**	16.Correctly performs the stages of admission, discharge and transfer of the patient	**Admit/discharge and transfer process**	**Vary good (4), good (3),Moderate(2),Poor (1),very poor (0)**	9- He/ she installs a suitable intravenous line for the patient and performs the necessary care	**IV line**	
0-5	Answer to questions about nursing (score : 0-5)process in Urology Cases		0-5	Answer to questions about Medical drugs in urology patients (score : 0-5)		**Evaluation of cognitive (10) skills**

Total score of Evaluation of Functional skills : 0-50
Total score of Evaluation of cognitive skills : 0-10
