# Supplementary material for: The effect of educational application in nursing internship clinical training on cognitive and functional skills and students’ satisfaction
Source: BMC Nurs. 2024 Jun 5;23:381. doi: 10.1186/s12912-024-01954-5 (PMC11151607; doi:10.1186/s12912-024-01954-5)
Supplement: Supplementary file 2 — Supplementary Material 2 [file 12912_2024_1954_MOESM2_ESM.pdf]

A satisfaction scale (adapted from Stokes, 2001)

N	Questions	Scale
1	I am able to access the mobile learning platform with an Internet connection to do my work.	
2	The resources I need are readily available through the mobile learning platform.	
3	I am satisfied with the degree of contact I have with my teacher when working through the mobile learning platform.	
4	I am pleased with the success I am having with completing my work through the mobile learning platform.	
5	My technology knowledge level is sufficient for learning through the mobile learning platform.	
6	I am feeling somewhat connected to the University setting by taking a class that places emphasis on learning through the mobile learning platform.	
7	I would prefer to take more of my classes through the mobile learning platform.	5 = very often<br>4 = often<br>3 = sometimes<br>2 = seldom<br>1 = never
8	Participating in the mobile learning platform has allowed me more flexibility in my daily activities.	
9	I would prefer more of the course materials in my traditional face-to-face classes to be in the mobile learning platform.	
10	I believe that working in the mobile learning platform enables me to play a more active role in the learning process.	
11	Communication with other students through the mobile learning platform is a positive experience.	
12	I find the mobile learning platform to be useful in helping me understand the material.	
13	The mobile learning platform is providing me with skills that I can use in other courses.	
14	I believe that the mobile learning platform is preparing me for future profession development.	
